# Supplementary figures and images for: Characterization of the NiRAN domain from RNA-dependent RNA polymerase provides insights into a potential therapeutic target against SARS-CoV-2
Source: PLoS Comput Biol. 2021 Sep 13;17(9):e1009384. doi: 10.1371/journal.pcbi.1009384 (PMC8478224; doi:10.1371/journal.pcbi.1009384)

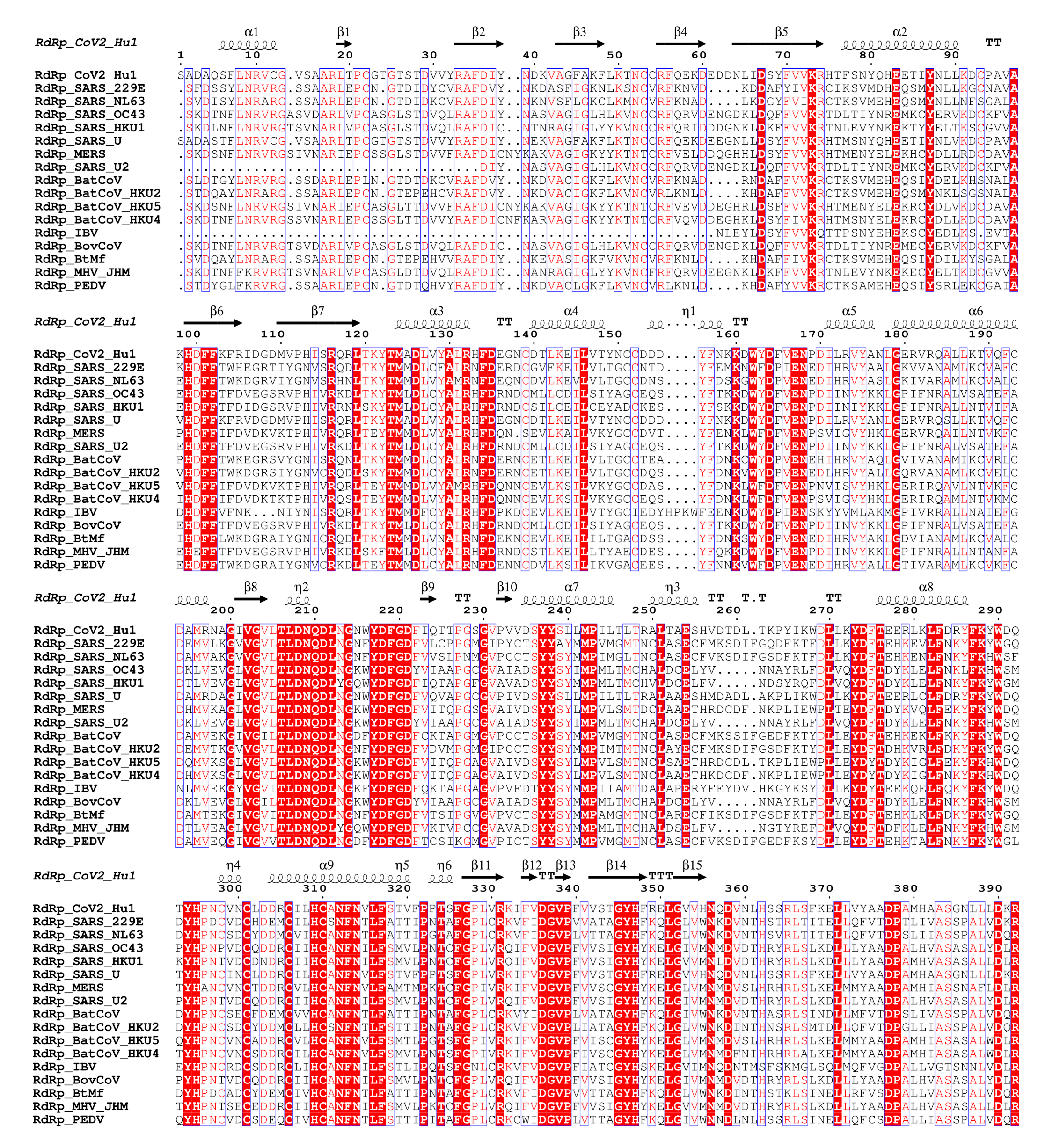

Supplement: S1 Fig — (TIF) [file pcbi.1009384.s001.tif]

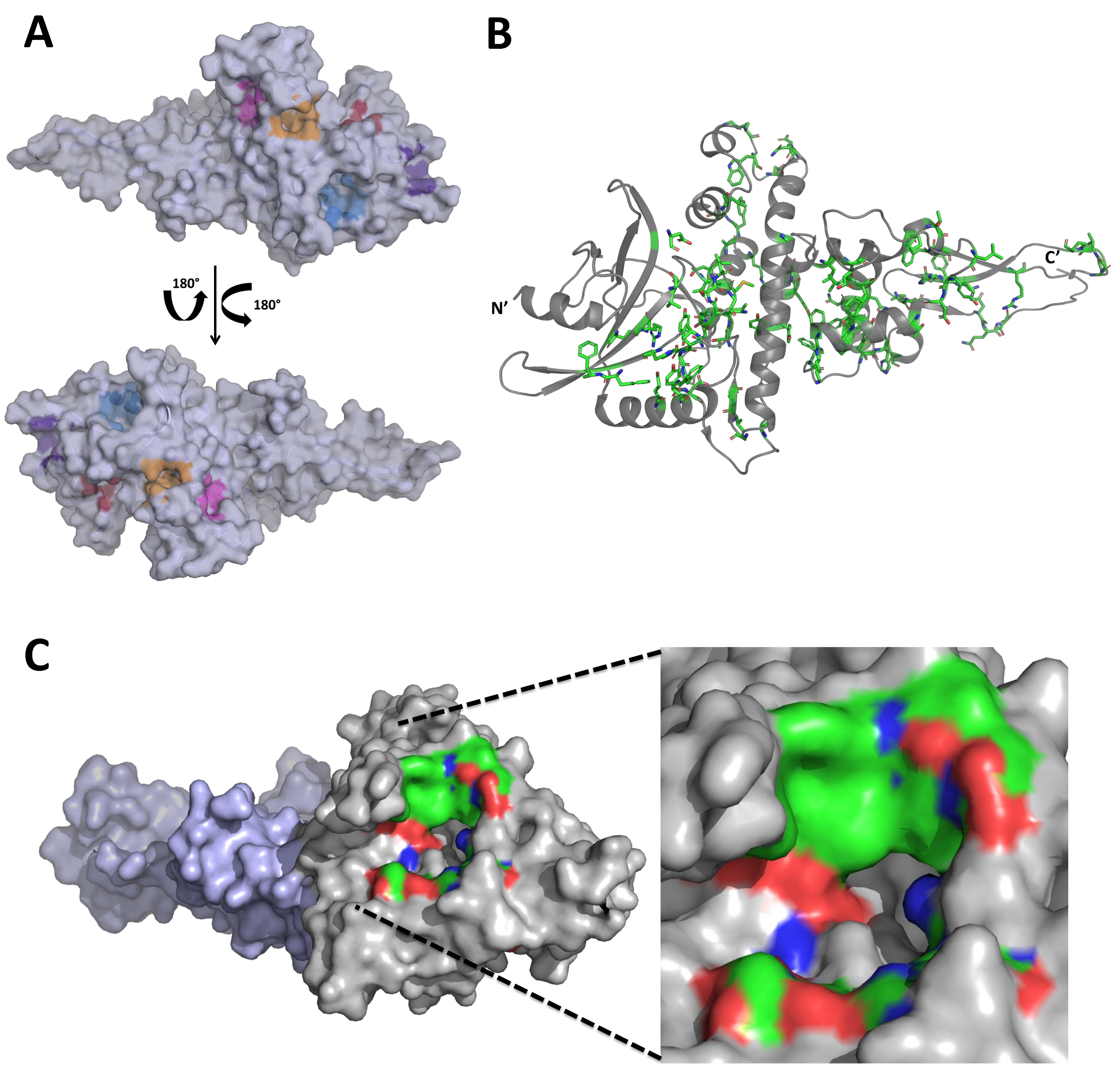

Supplement: S2 Fig — (A) Organization of the entry pockets at CoV-2-RdRp NiRAN domain lined with the strictly conserved residues. (B) While the conserved residues in the interface domain are scattered across the structural elements, the majority of the conserved residues in NiRAN domain lie between the antiparallel β-sheet and the immediately following helix bundle, possibly hinting at the NiRAN domain active site. (C) The predicted active site of the NiRAN domain possess both charged and uncharged residues, where in the charged residues primarily line the entry points of the pocket and the uncharged residues line in the deeper sections (Blue indicates positively charged regions, red indicates negatively charged regions and green indicates neutral regions, grey indicates regions beyond GTP-binding pocket). (TIF) [file pcbi.1009384.s002.tif]

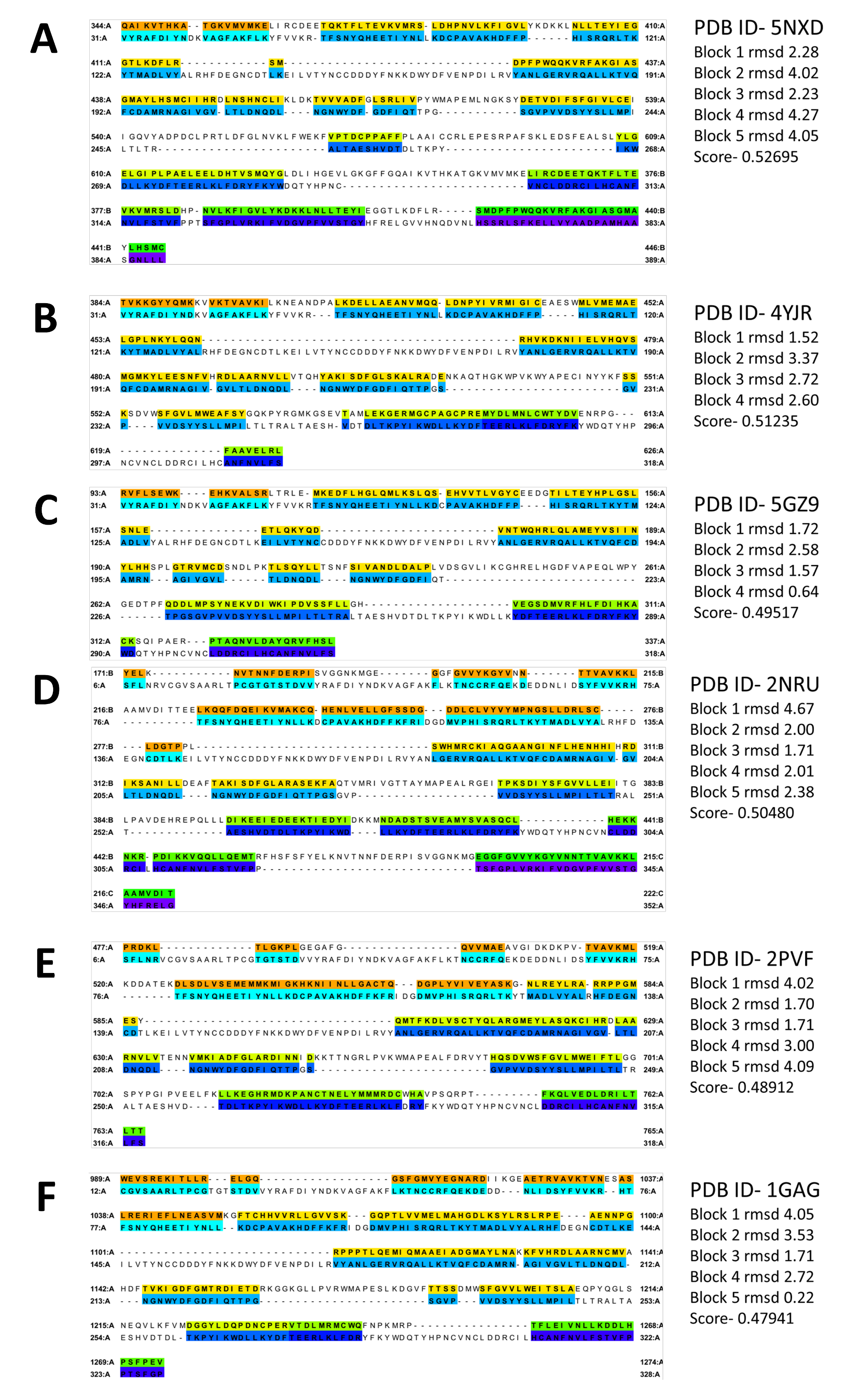

Supplement: S3 Fig — (A) Lim 2 kinase domain. (B) Syk kinase domain (C) O-mannosyl kinase domain. (D) IRAK4 kinase domain. (E) FGFR2 kinase domain. (F) Insulin receptor kinase domain. (Colour codes- Block 1: orange-cyan; Block 2: yellow-turquoise; Block 3: lime- deep blue; Block 4: green- deep blue; Block 5: green- purple). (TIF) [file pcbi.1009384.s003.tif]

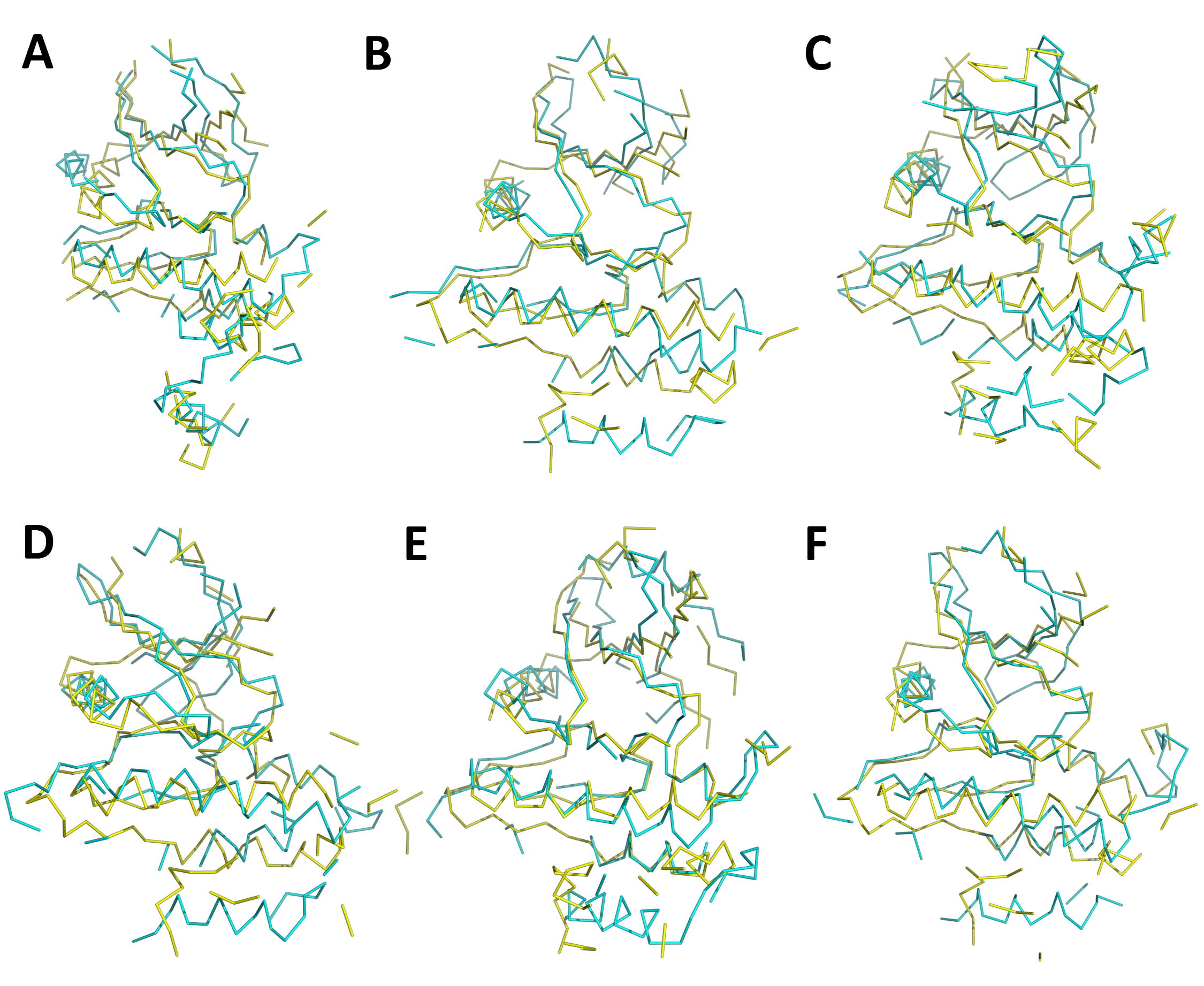

Supplement: S4 Fig — (A) Lim 2 kinase domain. (B) Syk kinase domain (C) O-mannosyl kinase domain. (D) IRAK4 kinase domain. (E) FGFR2 kinase domain. (F) Insulin receptor kinase domain. (The aforementioned kinases’ Cα chains are shown in cyan, CoV-2-RdRp NiRAN Cα chains are shown in yellow.) (TIF) [file pcbi.1009384.s004.tif]

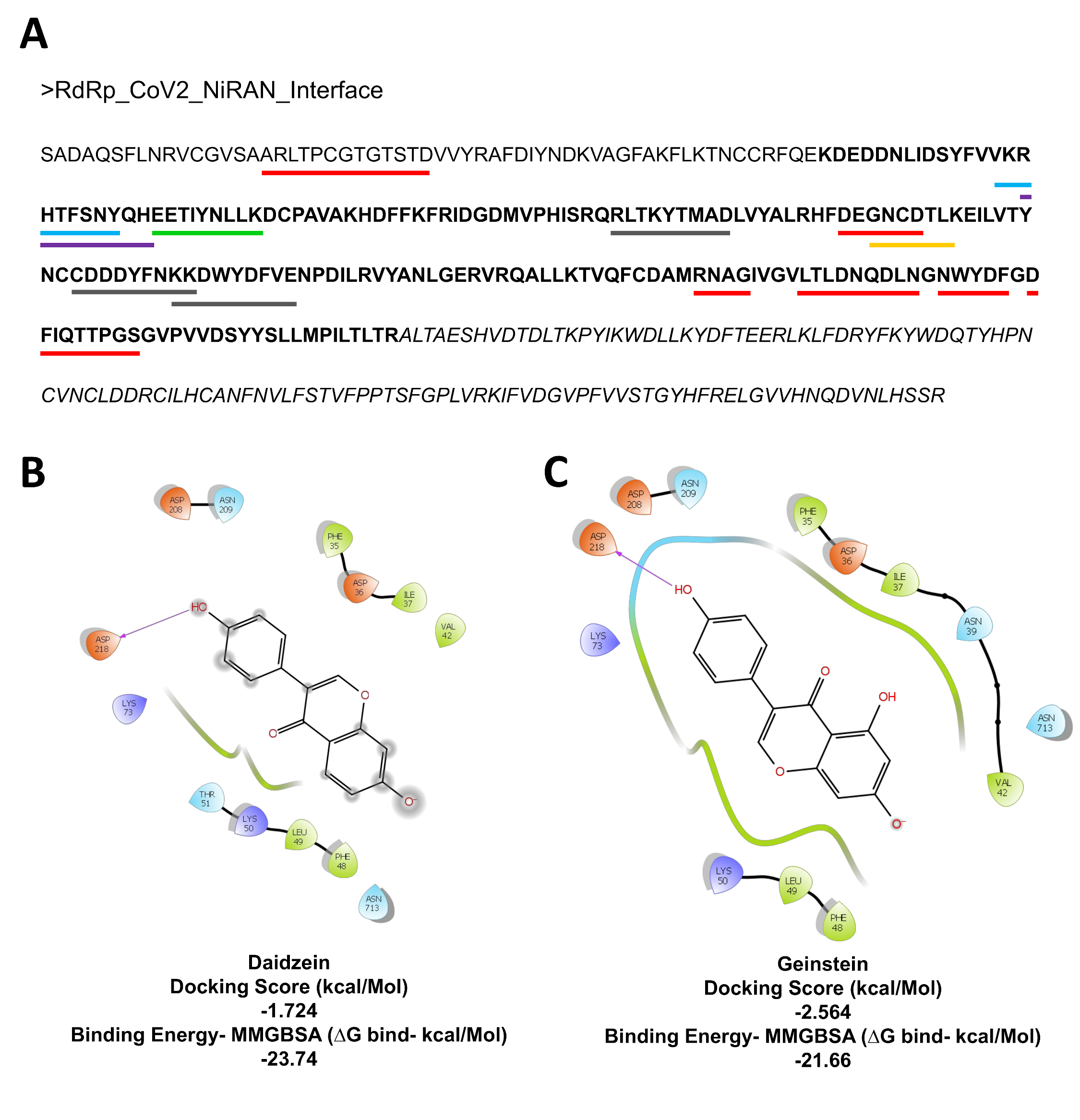

Supplement: S5 Fig — (A) Kinase consensus site search prediction also predicts multiple phosphorylation sites. (Bold- NiRAN domain; Italics- Interface domain; Underlines: Red- Kinase consensus sequence, Blue- PkC like motif, purple- PkA like motif, Green- Src kinase like motif, Grey- Unspecified Kinase like motifs, Yellow- Myristoylation site consensus sequence). (B) An inactive analog of Daidzein docked into the NiRAN domain putative active site. (C) An inactive analog of Geinstein docked into the NiRAN domain putative active site. (TIF) [file pcbi.1009384.s005.tif]

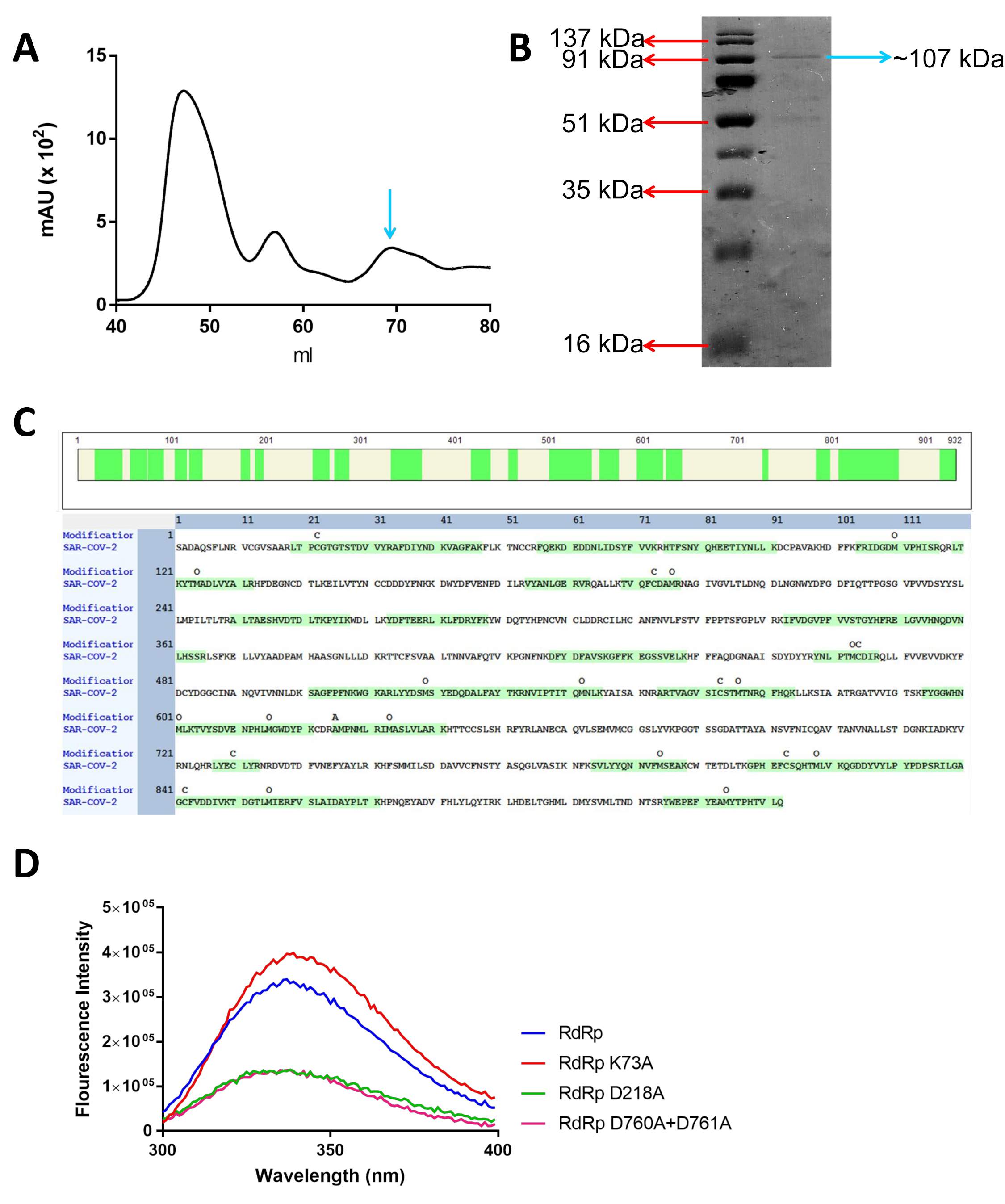

Supplement: S6 Fig — A) Size exclusion chromatogram of recombinant SARS-CoV-2 RdRp (The peak indicated with a blue arrow represents the purified protein sample). (B) SDS-PAGE profile of the purified SARS-CoV-2 RdRp (The band indicated with a blue arrow represents the purified protein sample and the molecular weight markers are indicated in red arrows). (C) Fluorescence spectra for native and mutant SARS-CoV-2 RdRp suggest that the incorporated mutations have negligible effect on the protein structural integrity. (TIF) [file pcbi.1009384.s006.tif]

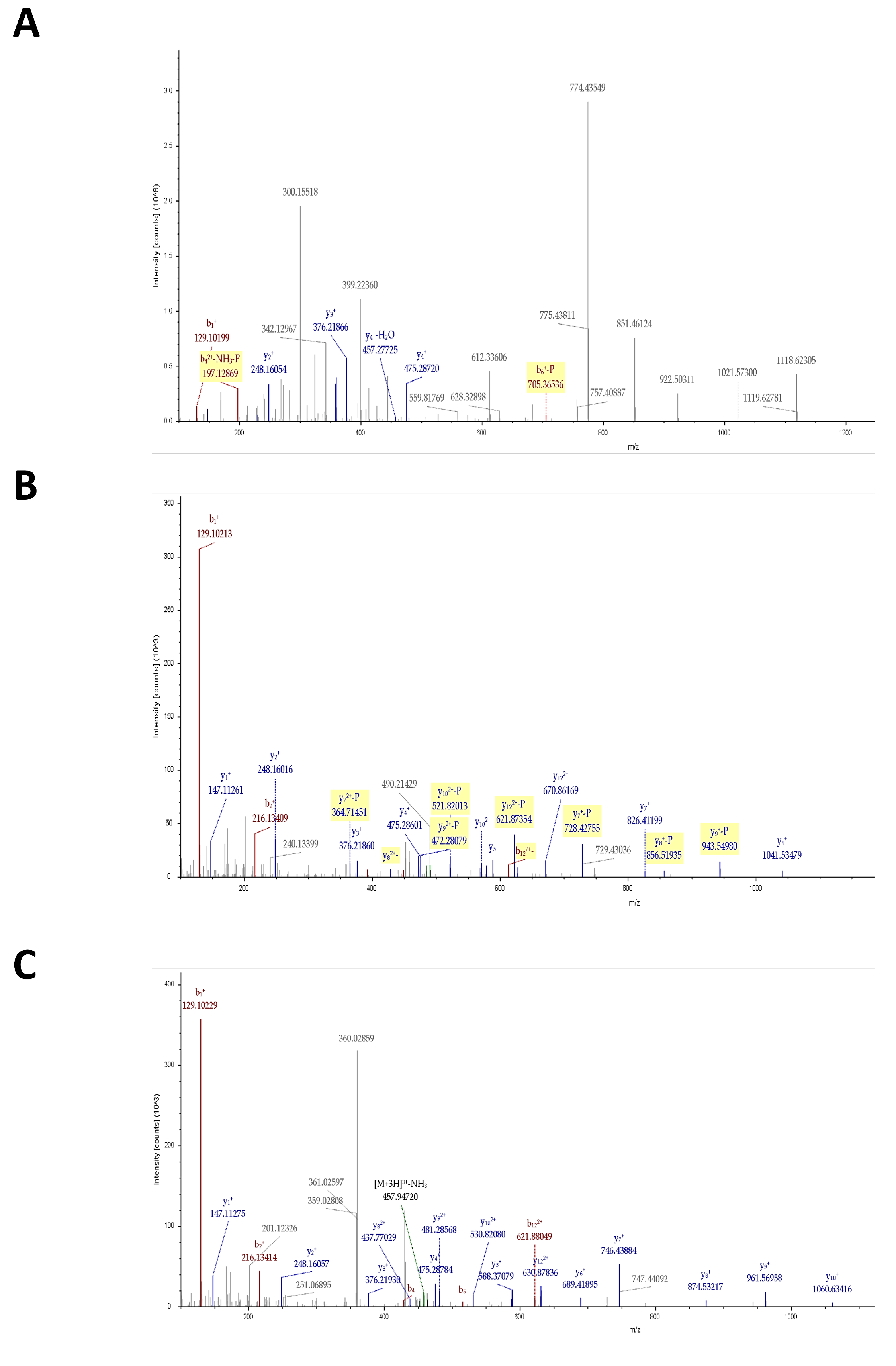

Supplement: S7 Fig — (A) Histone H1 treated with SARS-CoV-2 RdRp in presence of ATP and Mg2+. (B) Histone H1 treated with human Akt2 in presence of ATP and Mg2+. (C) Histone H1 treated with SARS-CoV-2 RdRp in the presence of Mg2+ only (The b and y ions are shown in red and blue, respectively). (TIF) [file pcbi.1009384.s007.tif]

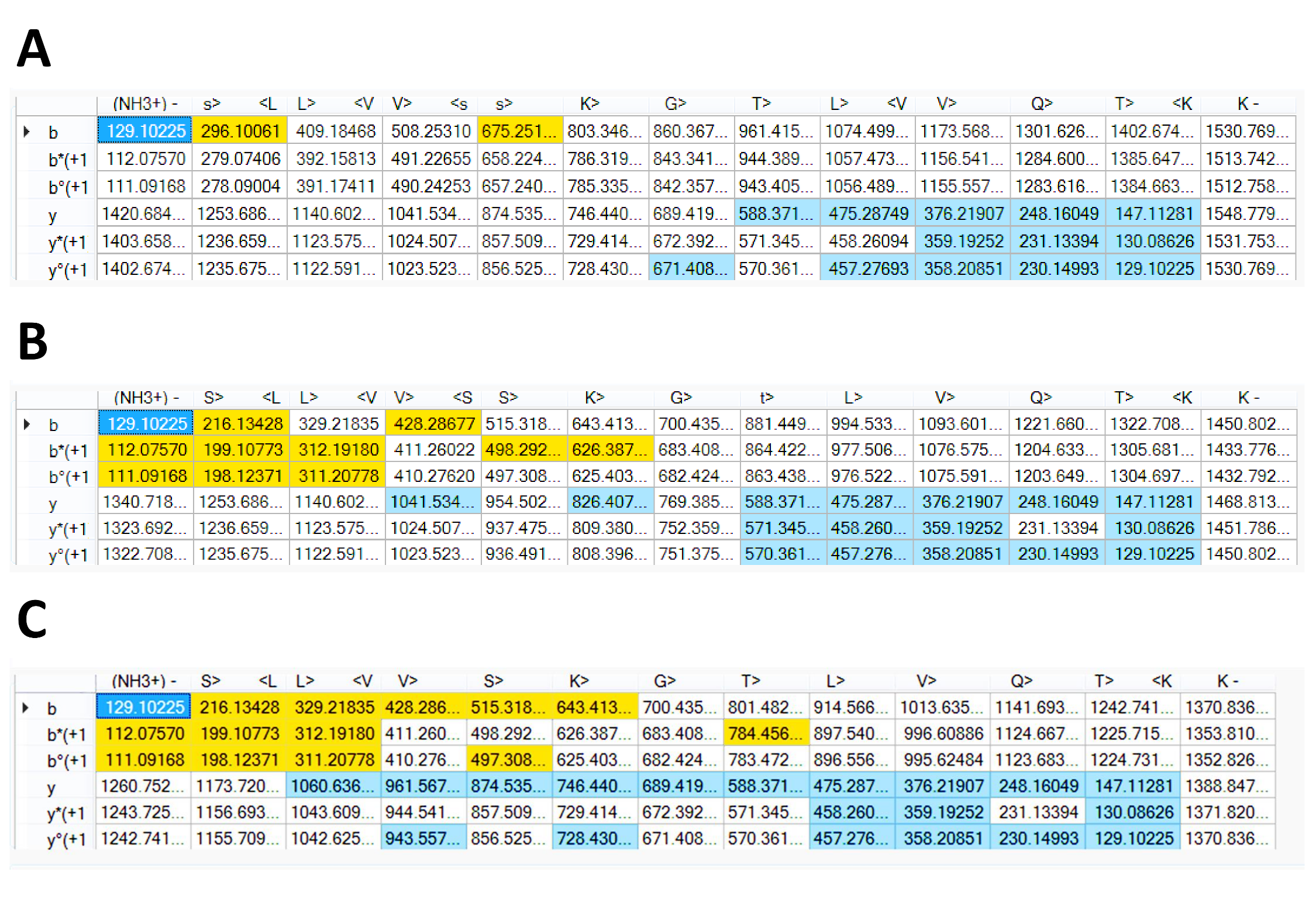

Supplement: S8 Fig — (A) Histone H1 treated with SARS-CoV-2 RdRp in presence of ATP and Mg2+. (B) Histone H1 treated with human Akt2 in presence of ATP and Mg2+. (C) Histone H1 treated with SARS-CoV-2 RdRp in the presence of Mg2+ only. (TIF) [file pcbi.1009384.s008.tif]
