# Supplementary material for: Characterization of the NiRAN domain from RNA-dependent RNA polymerase provides insights into a potential therapeutic target against SARS-CoV-2
Source: PLoS Comput Biol. 2021 Sep 13;17(9):e1009384. doi: 10.1371/journal.pcbi.1009384 (PMC8478224; doi:10.1371/journal.pcbi.1009384)
Supplement: Attachment — Submitted filename: Response_to_reviewers.docx [file pcbi.1009384.s018.docx]

**Response to Reviewers**

**Comments by the Editor:**

Structural analysis is used to suggest that the NiRAN domain of RNA-dependent RNA polymerase of CoV2 is a kinase. Then in silico docking is used to predict several small molecule drugs that might inhibit it. The manuscript is written very well. However, it completely lacks experimental data. Not even to support the proposition that it's a kinase. And in silico docking always detects seemingly good candidates, the vast majority of which end up having no real activity. Experiments would have to be included to cross the bar in PLOS Comp Biol.

**Authors’ Responses:**

**>>** We thank you for your comments. It greatly encouraged us to pursue this study further.

**>>** Over the past few months, we have cloned, over-expressed and purified the SARS-CoV-2RdRp/Nsp12. The identity of the purified protein was verified using mass spectrometry.

**>>** Using a luminescence based kinase assay system; we successfully show that the SARS-CoV-2 RdRp does indeed possess a kinase like activity; comparable to that of a known human kinase- Akt2.

**>>** In addition, we demonstrate that the kinase inhibitors Sorafenib, Sunitinib and SU6656 successfully inhibit the activity of both SARS-CoV-2 RdRp and Human Akt2.

**>>** We also show that of the top five compounds predicted to inhibit any kinase-like activity of SARS-CoV-2 RdRp, three compounds show significant inhibitory potentials**.**
