# Supplementary material for: Characterization of the NiRAN domain from RNA-dependent RNA polymerase provides insights into a potential therapeutic target against SARS-CoV-2
Source: PLoS Comput Biol. 2021 Sep 13;17(9):e1009384. doi: 10.1371/journal.pcbi.1009384 (PMC8478224; doi:10.1371/journal.pcbi.1009384)
Supplement: Attachment — Submitted filename: Response_to_reviewers.docx [file pcbi.1009384.s019.docx]

**Reviewer #1: (Authors’ responses are preceded by >> presented in bold font)**

The manuscript by Dwivedy et al. characterizes the RNA-dependent RNA polymerase from SARS-CoV-2 computationally and proposes an intriguing hypothesis that the NiRAN domain has “kinase like activity.” I cannot comment on the computational work, but as for the experimental portion there is one more important experiment lacking here, otherwise the authors cannot substantiate their claim about the activity. They measure ADP production from the RdRp using an ADP-Glo assay and conclude that there is kinase like activity. However, there are several problems with this conclusion. First, they merely measured ADP production, which implies ATP hydrolysis (or autophosphorylation), but does not show kinase-like activity. Secondly, they use the whole RdRp protein instead of the NiRAN domain. It seems possible that another part of the protein, say the RdRp active site, has ATPase activity.

**>> We thank you for the in-depth analysis of our study.**

**>> All changes incorporated in the manuscript and supplementary texts are highlighted in a yellow background.**

**>> The sub-sections in Results and Methods for the DFT calculations and the relevant figure(s) have been removed from the revised manuscript.**

They need to rule out this possibility, with one or more of the following experiments:

-purify the NiRAN domain separately and show ATP hydrolysis

**>> We attempted to either purify the NiRAN+Interface domain separately or cleave the NiRAN-Interface domain from the RdRp protein using Cathepsin D. However we failed to obtain a pure and functionally active NiRAN subunit.**

-show phosphotransfer activity for the whole protein or better the NiRAN domain. While no known substrates are known, standard generic substrates like dephosphorylated MBP should work.

**>> We utilized mass spectrometry based phosphoproteomic analysis to confirm the phosphotransfer enzymatic capability of SARS-CoV-2 RdRp.**

**>> We used dephosphorylated human Histone H1 as a phosphotransfer substrate and incubated the same with either SARS-CoV-2 RdRp or human Akt2 in presence of ATP and Mg^2+^. The following 7 sets of reactions were set up:**

**1. H1 + RdRp + ATP + Mg^2+^ (experimental protein reaction)**

**2. H1 + Akt2 + ATP + Mg^2+^ (kinase positive control)**

**3. H1 + BSA + ATP + Mg^2+^ (non-kinase ATP binding protein negative control)**

**4. H1 + RdRp + Mg^2+^ (experimental protein negative control)**

**5. H1 + Akt2 + Mg^2+^ (kinase negative control)**

**6. H1 + ATP + Mg^2+^ (self-phosphorylation negative control)**

**>> We observed phosphorylation in residues S86 and S89 in reaction 1 and in residue T92 in reaction 2. No phosphorylation was detected in other reactions.**

**>> Together these data suggest that SARS-CoV2-RdRp harbours a kinase/phosphotransferase like catalytic activity similar to serine-threonine kinases.**

**>> The relevant information is updated in the revised manuscript (Line- 287; Figures 5B, 5C, 5D; Supplementary Figures S7 and S8; Supplementary Table S8).**

-mutate residues in the NiRAN domain and show that the protein is still well folded but has a loss of ATP hydrolysis. They predict the active site of the NiRAN domain, but then need to test it out.

**>> To ascertain that the kinase/phosphotransferase activity observed using the ADP-Glo assay is harboured by the NiRAN domain of the SARS-CoV-2, we mutated two probable NiRAN active site residues K73 and D218 each to an alanine. Biochemical assay with the mutant proteins revealed a significant loss of activity with V_max_ reduced to nearly half as that of the wild type protein.**

**>> In addition, mutating the key aspartate dyad (D760 and D761) of the RdRp active site in the palm domain to alanines exhibited no effect on the kinase/phosphotransferase activity of SARS-CoV-2 RdRp.**

**>> These observations suggest that the kinase/phosphotransferase activity of SARS-CoV-2 RdRp is largely exhibited by the NiRAN domain. The relevant information is updated in the revised manuscript (Line- 275; Figures 5A; Supplementary Figures S6D).**

If not, then the authors can’t claim kinase like activity or any function to the NiRAN domain. While the potency of the kinase inhibitors is suggestive of kinase-like binding pocket, more needs to be shown to prove that.

**>> The mutational studies in combination with the mass spectrometry analysis clearly suggest that the NiRAN domain of the SARS-CoV-2 exhibits a kinase/phosphotransferase like activity capable of transferring a phosphate group to host proteins such as Histone H1.**

**Reviewer #2**: **(Authors’ responses are preceded by >> presented in bold font)**

This manuscript seeks to understand the function of the NiRAN domain of the SARS-CoV-2 polymerase, which is unique to nideoviruses. Based on both sequence and structural homology, the authors find that it is likely a kinase. In support of this hypothesis, they show it functions similarly to a known kinase, and that broad spectrum kinase inhibitors inhibit the activity of the NiRAN domain. Using docking, they also find some novel potential inhibitors. Together, their work provides valuable insight into this unique feature of the virus and starting points for drug discovery efforts.

**>> We thank you for the in-depth analysis of our study.**

**>> All changes incorporated in the manuscript and supplementary texts are highlighted in a yellow background.**

**>> The sub-sections in Results and Methods for the DFT calculations and the relevant figure(s) have been removed from the revised manuscript.**

Is there any evidence that the NiRAN domain is essential for the viral replication cycle? If not, it may not be a very useful drug target.

**>> To determine if the NiRAN domain’s function is essential for the viral replication cycle, we infected Vero E6 cells with SARS-CoV-2 and treated the infected cells with Sorafenib (an inhibitor of the NiRAN domain catalytic activity as shown in this study), either alone or in combination with the approved anti-COVID-19 drug, Remdesivir. We observed that Sorafenib is concentrations above 5 µM either alone or in combination with 1 µM Remdesivir effectively neutralized the virus.**

**>> The relevant information is updated in the revised manuscript (Line- 320; Figures 6C).**

**>> A recent study has shown the role of NiRAN domain in the formation of RNA G-cap during the viral RNA replication (Reference: *Cell.* 2021 Jan 7;184(1):184-193).**

**>> Also, another study shows that NiRAN domain also exhibits NMPylation catalytic activity on another SARS-CoV-2 viral protein, Nsp8. Interestingly, this NMPylation activity is exhibited primarily in the presence of Mn^2+^ and uses GTP and UTP as a substrate. However, the kinase/phosphotransferase activity is observed only the presence of ATP and Mg^2+^.**

**>> Taken together, this study and other recent literature highlight the essential role of NiRAN domain in sustaining a successful viral replication.**

Is the active site of the NiRAN domain sufficiently different from that of other kinases to potentially allow for specific inhibition of the virus without off-target effects?

**>> Kinase inhibitors such as Sorafenib and Sunitinib are approved drugs used in medical conditions such a hepatocellular and renal carcinomas. These drugs primarily inhibit the function of kinases associated with various cell division processes.** **As cancer cells undergo a much rapid cell divisions when compared to normal cells, they are generally more susceptible to these drugs than normal cells.**

**>> Interestingly most viral infections are known to cause mitotic arrest in the infected cells. Thus, we hypothesize that treatment of these infected cells with kinase inhibitors will have lesser effect on the cellular kinases, while specifically inhibiting the function of kinase like molecules harboured by the viruses, SARS-CoV-2 in this case.**

**>> This is further evident from our experiments where we treated SARS-CoV-2 infected Vero E6 cells with Sorafenib. While we observed a significant decline in the viral proliferation, no evident cellular death was detected.**

It would be useful to report docking scores and predicted binding affinities for a few suspected non-binders as a reference for judging the results for GTP, UTP, and potential inhibitors.

**>> In addition to ATP, GTP, UTP and the three kinases inhibitors (Sorafenib, Sunitinib and SU6656), we docked inactive analogs of kinase inhibitors (Daidzein and Geinstein) to the putative NiRAN active site. The docking scores for these molecules were -2.56 and -1.72 kcal/mol, which are lower than the active compounds docked into the putative NiRAN active site.**

**>> The relevant information is updated in the revised manuscript (Line- 215; Figures S5 B and C).**

Many experimental screens of FDA-approved drugs have been performed. Can the authors comment on why none of these kinase inhibitors showed up as hits? Is there still hope that the NiRAN domain could be targeted successfully?

**>> To the best of our knowledge, this is the first ever study that has performed docking of various anti-microbial compounds and kinase inhibitors specifically against the putative NiRAN domain active site.**

**>> Our primary aim for docking kinase inhibitors to the putative NiRAN active site was to decipher if NiRAN domain harbours any kinase/phosphotransferase like activity.**

**>> In addition, the 80 compounds that were docked into the putative NiRAN active site were selected from the available literature. These compounds target various viral and bacterial nucleotidyl-transferases and some of these compound are also FDA approved drugs.**

The figures are quite low resolution.

**>> In the revised submission, we have uploaded each Figure separately in high resolutions (300 + dpi).**

In Fig. 1, why not use the same color scheme in the structure in panel A and the secondary structure diagrams?

**>> We have updated Figure 1A (NiRAN structure) and 1B (NiRAN topology diagram) with the same colour scheme in the revised manuscript.**

**Reviewer #3:** **(Authors’ responses are preceded by >> presented in bold font)**

The manuscript by Dwivedy et al., characterizes the NiRAN domain of the RdRp protein from SARS-CoV-2 in terms of its structure, function, potential binding site and interactions with protease inhibitors. It is concluded that this domain possesses a kinase-like activity which could be inhibited by small ligands. It is therefore suggested that this domain may be a viable target for the development of new pharmaceuticals to fight of the COVID-19 pandemic.

Given the current state of the COVID-19 pandemic and the lack of therapies, any addition to the limited arsenal of tools to combat it, is important. From this perspective, the results presented in this manuscript are timely and relevant. Moreover, the manuscript is overall well written. However, there are multiple issues in the manuscript that need to be addressed before it is ready for publications. These are listed below:

**>> We thank you for the in-depth analysis of our study.**

**>> All changes incorporated in the manuscript and supplementary texts are highlighted in a yellow background.**

**>> The sub-sections in Results and Methods for the DFT calculations and the relevant figure(s) have been removed from the revised manuscript.**

Major points:

1. Line 139: Nothing that was discussed until this point actually supports the idea that the NiRAN domain assumes a kinase-like fold since its fold does not comply with the canonical kinase fold, nor with the other folds in Figure 1C.

**>> Prior to the comparison of the kinase like folds and the NiRAN domain fold, we performed a fold search using the NiRAN 3D structure using PDB as the target database. Both HHPred and ORION searches retrieved kinase and kinase like molecules in the top 500 hits, 15 of which are presented in Supplementary Tables 1 and 2.**

**>> The relevant information was presented in the Line 124 of the original manuscript and is available in the Line 125 of the revised manuscript.**

2. Line 205: The fact that the three kinase inhibitors docked into the NiRAN site is rather meaningless as are the docking scores. You would probably be able to dock into this site other compounds which are clearly not kinase inhibitors. A more convincing argument can be put forth by searching for inactive analogues of the kinase inhibitors and demonstrating that they don’t bind well into the site.

**>>** **Molecular docking studies employ the scoring functions to evaluate the docking modes of the ligands by conformational searches. These scoring functions simplify the calculations with many approximations to achieve high throughput screening. By using approximated scoring functions, molecular docking usually fails to accurately estimate binding energies when compared to experimental values. Therefore, the MMGBSA method is opted for rescoring as it is a fastest force-field based method that computes the free energy of binding, as compared to the other computational free energy methods, such as free energy perturbation (FEP) or thermodynamic integration (TI) methods. The MMGBSA method has been widely exploited in free energy calculations and MMGBSA rescoring overtly yields better results than traditional docking analysis.**

**>> For the docking analysis results presented in Supplementary Tables S3 and S5, we show both the docking score and the binding energy for the MMGBSA calculations. While the former is an indicator of the possibility of a ligand binding to a certain site on a protein, the latter is a more reliable representation of the strength of the binding.**

**>> The three identified kinase inhibitors Sunitinib, Sorafenib, and SU6656 against the NiRAN domain showed the docking scores of -3.5, -3.06, and -2.61 kcal/mol comparatively lesser than the ATP, GTP, and UTP.**

**>> However, the MMGBSA scores of three compounds (Sunitinib: -28.07, Sorafenib: -36.06, and SU6656: -25.07 kcal/mol) showed better values than the ATP (-17.84 kcal/mol), GTP (-15.77 kcal/mol), and UTP (-17.67 kcal/mol) molecules suggesting that the inhibitors may exhibit stronger binding affinities.**

**>> In addition to the three kinases inhibitors (Sorafenib, Sunitinib, and SU6656), we docked inactive analogs of kinase inhibitors (Daidzein and Geinstein) to the putative NiRAN active site. The docking scores for these molecules were -2.56 and -1.72 kcal/mol, respectively. The MMGBSA binding energy for these molecules were -23.74 and -21.66 kcal/mol, respectively; which are lower than the active compounds docked into the putative NiRAN active site.**

**>> The relevant information is updated in the revised manuscript (Line- 215; Figures S5 B and C).**

3. Line 214: Based on the above, I don’t think that the docking analysis supports the kinase / phosphotransferase activity of NiRAN.

**>> In the revised manuscript, the mutational studies in combination with the mass spectrometry analysis clearly suggest that the NiRAN domain of the SARS-CoV-2 exhibits a kinase/phosphotransferase like activity capable of transferring a phosphate group to host proteins such as Histone H1.**

**>> The relevant information is updated in the revised manuscript (Line- 275 and 287; Figures 5A, 5B, 5C, 5D; Supplementary Figures S7 and S8; Supplementary Table S8)**

4. Line 251: I fail to see the value of the DFT calculations. The HOMO and LUMO energies by themselves are meaningless as are the corresponding energy differences. Similarly, the electrostatic potentials don’t add anything to the discussion. They may have been interesting had the authors tried to use them to explain the resulting binding modes, for example, by looking at how they match the electrostatic potential of the site, but this was not attempted.

5. Line 263: Why does the fact that the HOMO is more spread than the LUMO suggestive of a possible nucleophilic reaction? I would think that this spreading would reduce the electronic density at each point thereby reducing the strength of a nucleophilic attack. And which nucleophilic reaction are we talking about in the first place?

6. Line 280: Chemical reactivity towards what?

**>> As we have presented the biochemical efficacies of the inhibitors against the proposed kinase like activity of the SARS-CoV-2 RdRp, we agree that DFT analysis fails to add any further significance.**

**>> Thus, the sub-sections in Results and Methods for the DFT calculations and the relevant figure(s) have been removed from the revised manuscript.**

7. Line 350: Which simulations are we talking about? If this refers to the MM-GBSA simulations then their results were not discussed in the manuscript and furthermore, I don’t see what they add.

**>> Kindly refer to our response to Point no. 2 above.**

8. Line 352: Technically, 1000 nM count as nano-molar but these are really micro-molar concentrations.

**>> As majority of the available literature presents the IC_50_ and K_i_ values of kinase inhibitors in nanomolar concentration (range is ~200-800 nM), we chose to present a similar nomenclature.**

9. Materials and methods: The docking protocol was not validated in any way. Table S3 contains a list of compounds selected for docking. Some of these compounds were solved in complex with their corresponding targets. The authors should try and reproduce the binding modes of these compounds with their docking protocol. Without this, how can we tell that the docking protocol is suitable for the task in hand?

**>> In order to validate the docking and MMGBSA calculation protocols, we chose the crystal structure of Zika virus Methyl-transferase co-crystallized with Sinefungin (PDB ID- 5MRK). We retrieved the PDB file and manually removed the coordinates for Sinefungin, to generate the PDB file for the Methyl-transferase.**

**>> The 3D coordinates for Sinefungin were retrieved from PubChem and docked into the Methyl-transferase structure, which was followed by MMGBSA calculations.**

**>> In the crystal structure, the Sinefungin is bound in the S-adenosyl-methionine binding pocket and forms 8 H-bonds with Ser56, Gly86, Trp87, Lys105, His110, Glu111, Asp131, and Asp146 (Kindly see the figure below; Reference- *Arch Virol.* 2017 Jul;162(7):2091-2096.).**

**>> Our docking and MMGBSA analysis revealed that Sinefungin forms 8 H-bonds with Ser56, Gly86, Trp87, Lys105, Glu111, Asp131, Val132, and Asp146; thus validating the docking and MMGBSA calculation protocols (Docking score: -9.445 kcal/mol; MMGBSA binding energy: -67.51 kcal/mol) (Kindly see the figure below).**

**>> The docked and co-crystallized Sinefungin molecules superimposed perfectly within the S-adenosyl-methionine binding pocket of Zika virus Methyl-transferase (Kindly see the figure below; crystal structure Sinefungin in raspberry backbone and docked Sinefungin in orange backbone).**

**>> While the details of this validation protocol are not presented in the manuscript, it is briefly mentioned in the updated manuscript (Line- 443).**

10. Line 405: As stated above, what is the purpose of the MM-GBSA calculations?

**>> Kindly refer to our response to Point no. 2 above.**

11. Line 424: As noted above, I don’t see the value of the DFT calculations. Moreover, an implicit solvent model can hardly be qualified as “near physiological state” (line 431). Also, what were the starting geometries for the DFT calculations?

**>> Kindly refer to our response to Point no. 4, 5 and 6 above.**

12. Lines 525 and 535: Docking scores by themselves are rather meaningless and are only good for comparison purposes. Furthermore, the docking scores of GTP and UTP were found to be -9.84 and -6.59 kcal/mol, respectively. Those of the three broad spectrum inhibitors were found to range between -2.6 and -3.5 kcal/mol. How could the latter then be classified as having “significantly low free binding energies”?

**>> Kindly refer to our response to Point no. 2 above.**

Minor points:

1. Line 72 and on: PDB codes should be given when discussing the cryo-EM structures.

**>> We have tried to incorporate all the PDB IDs for the relevant structures as and when they are referred to in the text.**

2. Line 107: The sentence starting with “Apart from” is not clear. Are there 78 or 84 conserved residues?

**>> There are a total of 84 conserved residues.**

**>>This sentence has been appropriate rephrased in the revised manuscript (Line- 109).**

3. Line 114: What is the meaning of “entry” pockets and how does the number “five” relate to the single site discussed later in the work?

**>> While attempting to map the putative active site of the NiRAN domain, we chose an out-in approach and followed the path inwards of the entry pores. NiRAN is known to bind nucleotide triphosphates, divalent cations, proteins and growing RNA chains. These substrates possibly follow different entry pores and subsequently meet at the active site. Our mapping of the NiRAN domain surface revealed five possible sites of entry, two of which remain buried between the NiRAN and the palm domains, and two of the remaining pores eventually meet at what we propose as the NiRAN domain active site.**

**>> Entry pockets play a critical role in the biochemical activity of an enzyme. In addition to being the entry/exit routes for substrates/co-factors/ions, these pockets also define the location of the possible active site of an enzyme. For example, in bacterioferritins, the B-pore serves as the entry pore for Fe^2+^ which facilitates its movement to the di-nuclear centre. Similarly, the 4-fold and 3-fold axis pores of bacterioferritins serve as the exit route for Fe^3+^ out from the location of the Heme moiety. (Reference 1: *Acc Chem Res.* 2017 Feb 21; 50(2): 331–340. Reference 2: *Chem Rev.* 2015 Jan 14;115(1):295-326.)**

4. Line 132: Change “comprises of an” to “comprises an”.

**>> The suggested change has been incorporated in the revised manuscript (Line- 133).**

5. Line 184: This seems like a repeat of what was already discussed on line 175.

**>> In the revised manuscript, one of these two sentences, corresponding to Line 175 of the original manuscript has been removed.**

6. Line 213: I think it should be Table S4 rather than Table S3.

**>> The Supplementary Tables have been updated in the revised manuscript.**

7. Line 339: Change “fewer” to “less”.

**>> The suggested change has been incorporated in the revised manuscript (Line- 359).**

8. Line 355: Change “Sunitinib as” to “Sunitinib were”.

**>> The suggested change has been incorporated in the revised manuscript (Line- 375).**

9. Line 413: The “.” Should be moved to after the “)”.

**>> The suggested change has been incorporated in the revised manuscript (Line- 442).**
