# Supplementary material for: Characterization of the NiRAN domain from RNA-dependent RNA polymerase provides insights into a potential therapeutic target against SARS-CoV-2
Source: PLoS Comput Biol. 2021 Sep 13;17(9):e1009384. doi: 10.1371/journal.pcbi.1009384 (PMC8478224; doi:10.1371/journal.pcbi.1009384)
Supplement: Attachment — Submitted filename: Response_to_reviewers.docx [file pcbi.1009384.s020.docx]

**Reviewer #3:** **(Authors’ responses are preceded by >> presented in bold font)**

Reviewer #3: The authors have adequately responded to all my comments on the original submission and I now find the manuscript ready for publication. Still, I have found a few typos that should be corrected. These are listed below:

**>> We sincerely thank you for reviewing our study.**

1. Line 47: “play” -> “playing”

**>> The correction has been duly incorporated in the revised manuscript. (Line 57)**

2. Line 85: “hinting as” -> “hinting at”

**>> The correction has been duly incorporated in the revised manuscript. (Line 85)**

3. Line 127: “4” -> “four”

**>> The correction has been duly incorporated in the revised manuscript. (Line 127)**

4. Line 158: “retrieved in” -> “retrieved by”

**>> The correction has been duly incorporated in the revised manuscript. (Line 158)**

5. Line 217: “6” -> “six”

**>> The correction has been duly incorporated in the revised manuscript. (Line 218)**

6. Line 252: “docking in” -> “docking”

**>> The correction has been duly incorporated in the revised manuscript. (Line 253)**

7. Line 299: “like of” -> “type”

**>> The correction has been duly incorporated in the revised manuscript. (Line 300)**

8. Line 303: “in appropriate” -> “under appropriate”

**>> The correction has been duly incorporated in the revised manuscript. (Line 304)**

9. Line 362: “spread across” -> “spreads across”

**>> The sentence containing the aforementioned phrase has been restructured as: “With a basic reproduction number ranging from 1.4 to 3.9, the disease has quickly disseminated across the globe within the past year”, in the revised manuscript. (Line 362)**

10. Line 399: “such Rift” -> “such as Rift”

**>> The correction has been duly incorporated in the revised manuscript. (Line 400)**

11. Line 616: “present” -> “presents”

**>> The correction has been duly incorporated in the revised manuscript. (Line 617)**

12. Line 648: “interaction” -> “interactions”

**>> The correction has been duly incorporated in the revised manuscript. (Line 649)**
